# Approaches for retraining sEMG classifiers for upper-limb prostheses

**DOI:** 10.3389/fnbot.2025.1627872

**Published:** 2025-10-01

**Authors:** Tom Donnelly, Elena Seminati, Benjamin Metcalfe

**Affiliations:** ^1^Centre for Accountable, Responsible, and Transparent AI (ART-AI), Department of Computer Science, University of Bath, Bath, United Kingdom; ^2^Bath Institute for the Augmented Human, University of Bath, Bath, United Kingdom; ^3^Department for Health, University of Bath, Bath, United Kingdom; ^4^Department of Electronic and Electrical Engineering, University of Bath, Bath, United Kingdom

**Keywords:** surface electromyography, hand gesture recognition, inter-session retraining, machine learning, myoelectric prostheses

## Abstract

**Introduction:**

Abandonment rates for myoelectric upper limb prostheses can reach 44%, negatively affecting quality of life and increasing the risk of injury due to compensatory movements. Traditional myoelectric prostheses rely on conventional signal processing for the detection and classification of movement intentions, whereas machine learning offers more robust and complex control through pattern recognition. However, the non-stationary nature of surface electromyogram signals and their day-to-day variations significantly degrade the classification performance of machine learning algorithms. Although single-session classification accuracies exceeding 99% have been reported for 8-class datasets, multisession accuracies typically decrease by 23% between morning and afternoon sessions. Retraining or adaptation can mitigate this accuracy loss.

**Methods:**

This study evaluates three paradigms for retraining a machine learning-based classifier: confidence scores, nearest neighbour window assessment, and a novel signal-to-noise ratio-based approach.

**Results:**

The results show that all paradigms improve accuracy against no retraining, with the nearest neighbour and signal-to-noise ratio methods showing an average improvement 5% in accuracy over the confidence-based approach.

**Discussion:**

The effectiveness of each paradigm is assessed based on intersession accuracy across 10 sessions recorded over 5 days using the NinaPro 6 dataset.

## 1 Introduction

The need for upper-limb prostheses continues to rise, and in 2021, there was an estimated 1.5 million upper-limb amputees worldwide (Nazarpour et al., 2021). Concurrently, prosthesis abandonment rates remain as high as 44% in 2020; abandonment is problematic and can lead to further injury as a result of compensatory movements of intact limbs (Salminger et al., 2022; Rausch et al., 2022). There are many reasons for device abandonment, but key drivers include weight, lack of sensory feedback, and functional limitations (such as unexpected movements and range of movements limited by simplistic control strategies; Ostlie et al., 2012; Cordella et al., 2016). A 2016 review of upper-limb amputee needs showed that improved functionality was critical and specifically identified: increased individual control of actuators, increased number of movements, and dexterity improvements such as control of the grip strength and precision of actuation (Cordella et al., 2016).

**Figure 1:**
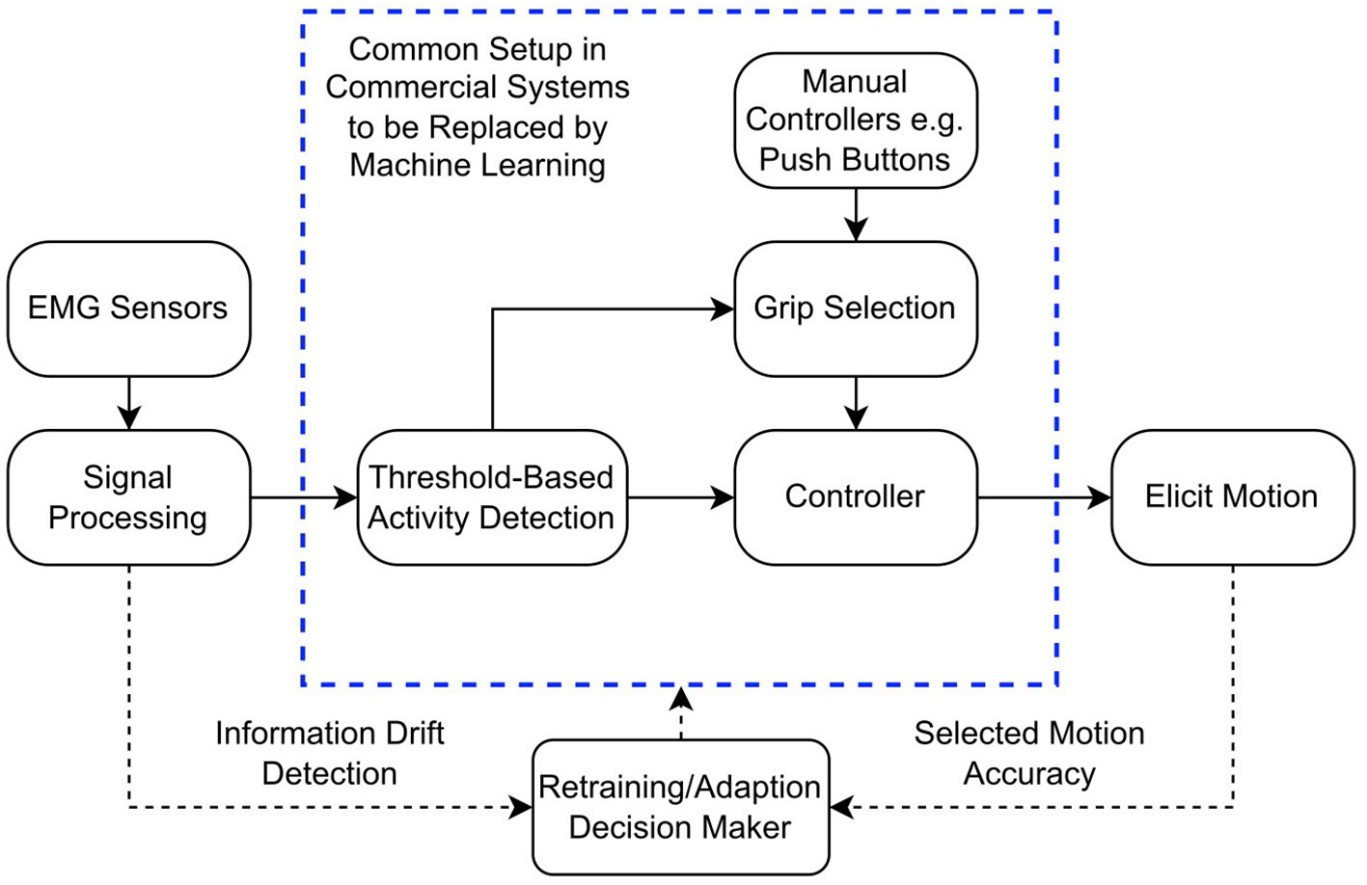
Block diagram demonstrating the typical myoelectric prosthesis controller. The dashed bounding box represents the parts of the control system that could be replaced by machine learning algorithms, reducing the input to just the EMG sensors. The dotted lines represent how retraining could be implemented in the controller.

To limit the computational demand and weight of the prosthesis controller, typical myoelectric prostheses use gross muscle activations (recorded from residual muscles in the stump) as inputs. These control the movement of the fingers, whilst the wrist and thumb positions are changed manually (Calado et al., 2019). Pattern recognition algorithms have been used to provide simultaneous control of multiple degrees-of-freedom, using machine learning algorithms to classify movement intention from surface electromyographic (sEMG) signals (Igual et al., 2019). Figure 1 shows a typical myoelectric prosthesis control system and illustrates where machine learning might be implemented. Machine learning classifiers have achieved accuracies as high as 99.52% on a 15-class sEMG dataset using 8 sEMG channels, demonstrating the potential for significant improvement of control by expanding the degrees-of-freedom (Bhagwat and Mukherji, 2020).

Despite the potential benefits of pattern recognition schemes, commercial adaptation remains limited. Barriers include computational complexity arising from machine learning and the need for a greater number of sEMG electrodes (Atzori and Müller, 2015; Parajuli et al., 2019).

Electromyographic signals recorded using sEMG are non-stationary (Phinyomark et al., 2012); electrode location (Hargrove et al., 2006), limb position (Scheme et al., 2010), temperature (Racinais, 2013), muscle fatigue (Guo et al., 2017), and skin-electrode impedance (Roy et al., 2007; Sae-lim et al., 2018) all influence the recorded signal. Noise and interference are ever present from sources including power lines and muscle crosstalk (Chen et al., 2023; Germer et al., 2021). Many of these factors are either constrained or minimised in a controlled laboratory environment. However, in a home-use setting, they can contribute dramatically to a decrease in sEMG signal quality and thus classifier performance. For example, the reduction in classification accuracy between a morning training session and an afternoon testing session can be as high as 27% (mean over 10 participants; Palermo et al., 2017). Accounting for the non-stationarity of sEMG within sessions, such as from limb position changes, can be achieved by training a classifier in multiple positions and applying sensor fusion (Fougner et al., 2011; Mobarak et al., 2025).

Similarly, retraining or adapting classifiers on data from sessions across multiple days is a potential mechanism to enable translation from the laboratory to the home. Retraining can involve creating a new classifier by combining seen and new windows of sEMG or updating an existing classifier through incremental training (Radmand et al., 2014). In contrast, adaption updates the model's hyper-parameters directly based on some external input (Vidovic et al., 2016; Huang et al., 2017). Adaptation based on new data has been shown to improve classification accuracy by up to 25% (Vidovic et al., 2016), whilst retraining using data from different limb positions increases accuracy across all positions by approximately 13% (Radmand et al., 2014).

Instead of using *all* new windows in retraining it is possible to select only the most beneficial windows. This reduces computational complexity by only retraining on windows that are likely to improve accuracy. Sensinger et al. showed that offline supervised window selection increased accuracy on unseen data by 30% whilst offline unsupervised methods increased accuracy by 20% (Sensinger et al., 2009). Online retraining has also been demonstrated by comparing an iterative learner to a non-adaptive one, with a 9.03% reduction in accuracy degradation at the end of 1 day (Huang et al., 2017). Online retraining has the advantage that it may facilitate continual adaptation, albeit at the cost of additional computational complexity. Alternatively, instead of retraining the classifier, it is possible to simply reject poor-quality windows of testing data using a confidence score from the classifier. This has been shown to reduce unintentional movements and increase classifier accuracy but comes with the risk of unresponsiveness if too many windows are rejected (Scheme et al., 2013; Robertson et al., 2019).

The selection of optimal windows for retraining provides a distinct benefit for adapting sEMG classifiers between independent recording sessions. However, few methods exist for directly selecting optimal data and comparison of the respective benefits and drawbacks of existing paradigms is limited. This study provides a comparison of two existing window selection paradigms from the literature, supervised confidence-based (Sensinger et al., 2009) and Edited Nearest Neighbour (ENN; Ding et al., 2019). A third, quality acceptance (QA), is additionally compared and is novel, using the signal-to-noise ratio (SNR) of each window. The confidence-based paradigm was selected to provide a retraining option driven by the output of the current classifier trained on previous windows. The ENN paradigm alternatively explores the new windows independently to the pattern recognition classifier, using a nearest neighbour algorithm. The proposed QA paradigm accepts windows above a signal quality threshold and is thus driven solely by the prior conception of an *acceptable* signal. The selected paradigms provide a range of complexity in their decision-making, allowing comparison of the underlying selection processes in addition to their performance.

The impact of the paradigms on classification accuracy is evaluated on an open source dataset, Ninapro DB6, which contains data obtained from 10 participants (each over 10 independent recording sessions; Palermo et al., 2017). As such, this work presents the first comparison, to the authors' knowledge, between any of the paradigms on multi-session data obtained over several days. All three paradigms were assessed for retraining, rejection, and a combination approach. Furthermore, the complexity of the paradigms and their capacity to perform on noisier data are assessed, highlighting their suitability to clinical applications. In addition, the assessments are performed using two common sEMG classifiers, linear discriminant analysis (LDA), a traditional classifier, and convolutional neural networks (CNN), which allows iterative training. This enables comparison of the impact of the paradigms on each classifier and the comparison of the individual classifier's application to retraining.

The study is organised with the methods presented in Section 2, detailing the data used, the paradigms, and the metrics for comparison. The results are presented in Section 3. An analysis and discussion of the paradigm performance results is provided in Section 4, additionally identifying the benefits and drawbacks of the compared paradigms.

## 2 Methods

### 2.1 Datasets

To assess the proposed window selection paradigms, the Non-Invasive Adaptive Hand Prosthetics Database 6 (DB6) was used (Palermo et al., 2017). DB6 is an open dataset of 10 healthy anatomically intact participants who performed a series of recording sessions twice daily for 5 consecutive days, for 10 total sessions per participant. In each session, the participants performed 12 repetitions of 7 different grasp activations in order: Large Diameter, Adducted Thumb, Index Finger Extension, Medium Wrap, Writing Tripod, Power Sphere, and Precision Sphere. This presents an 8-class problem with the inclusion of the rest periods as class zero. Each grasp activation involved moving from rest, picking up an object suitably shaped for the grasp, holding it for 4 s, and then placing the object back down. Each participant was guided to perform each 4-s activation, with a 4-s rest between individual grasps. sEMG signals were recorded using 14 Delsys Tringo Wireless electrodes (Delsys Inc, USA) sampled at 2 kHz. Electrodes were placed circularly at 2 positions of the forearm. One circle of 8 electrodes was placed around the radio-humeral joint, and the other of 6 was placed below the first. The dataset's baseline accuracy score of 52.43% was established using the Waveform Length feature and a Random Forest classifier (Palermo et al., 2017).

### 2.2 Pre-processing

Pre-processing consisted of min-max normalisation of the sEMG between -1 and 1, which preserves the shape and distribution of the signals whilst bounding it to a set range that is more suitable for use as CNN inputs. A sliding window approach was used to segment the signal, using a 200 ms window width and 10 ms step. These values are recommended for real-time classification of sEMG (Hakonen et al., 2015). Time-domain features were then extracted from each window (waveform length, zero crossings, slope sign change, mean absolute value; Hudgins et al., 1993). Classes were assigned to each window based on the *restimulus* vector provided in DB6. This vector was created post-recording to identify the true periods of sEMG activity from the participant, which typically last longer than the originally prompted 4 s.

Whilst the *restimulus* vector provided in DB6 differentiates between clear activity and rest, in some cases rest periods are contaminated with sEMG activity. Contaminants are likely the result of small adjustments made by the participant following the grasp. Therefore, some rest periods are significantly shorter than 4 s.

A full second of clean rest was found per recording for the SNR calculation. The duration of each rest period was assessed in the *restimulus* vector. Where a marked rest period was found to be longer than 2 s, the central 1 s was used for that signal's SNR calculation. Table 1 presents the equations for all features extracted from each window.

**Table 1:** Equations for each feature used for the machine learning algorithms, and the SNR for the QA paradigm.

**Feature**	**Equation**
Mean absolute value	$\frac{\overset{N}{\underset{i=0}{\sum }}|x_{i}|}{N}$
Slope sign changes	$\begin{array}{c} \overset{N-1}{\underset{i=1}{\sum }}f\left(x\right) \end{array}$
	$f(x)=\left\{ \begin{array}{l} 1\;(x_{i}>x_{i+1}\;x_{i}>x_{i-1})\;|\;(x_{i}<x_{i+1}\;x_{i}<x_{i-1})\\ 0\text{ otherwise} \end{array}\right.$
Zero crossings	$\begin{array}{c} \overset{N-1}{\underset{i=0}{\sum }}f\left(x\right) \end{array}$
	$f(x)=\left\{ \begin{array}{l} 1\;(x_{i}>0\;x_{i+1}<0)\;|\;(x_{i}<0\;x_{i+1}>0)\\ 1\text{ otherwise} \end{array}\right.$
Waveform length	$\overset{N-1}{\underset{i=0}{\sum }}|x_{i+1}-x_{i}|$
SNR	10log_10_(*P*_*s*_/*P*_*r*_)

*P*_*s*_ is the sum of the Fast Fourier Transform (FFT) of the signal period, and *P*_*r*_ is the sum of the FFT of the first acceptable rest period of at least 1 s.

**Figure 2:**
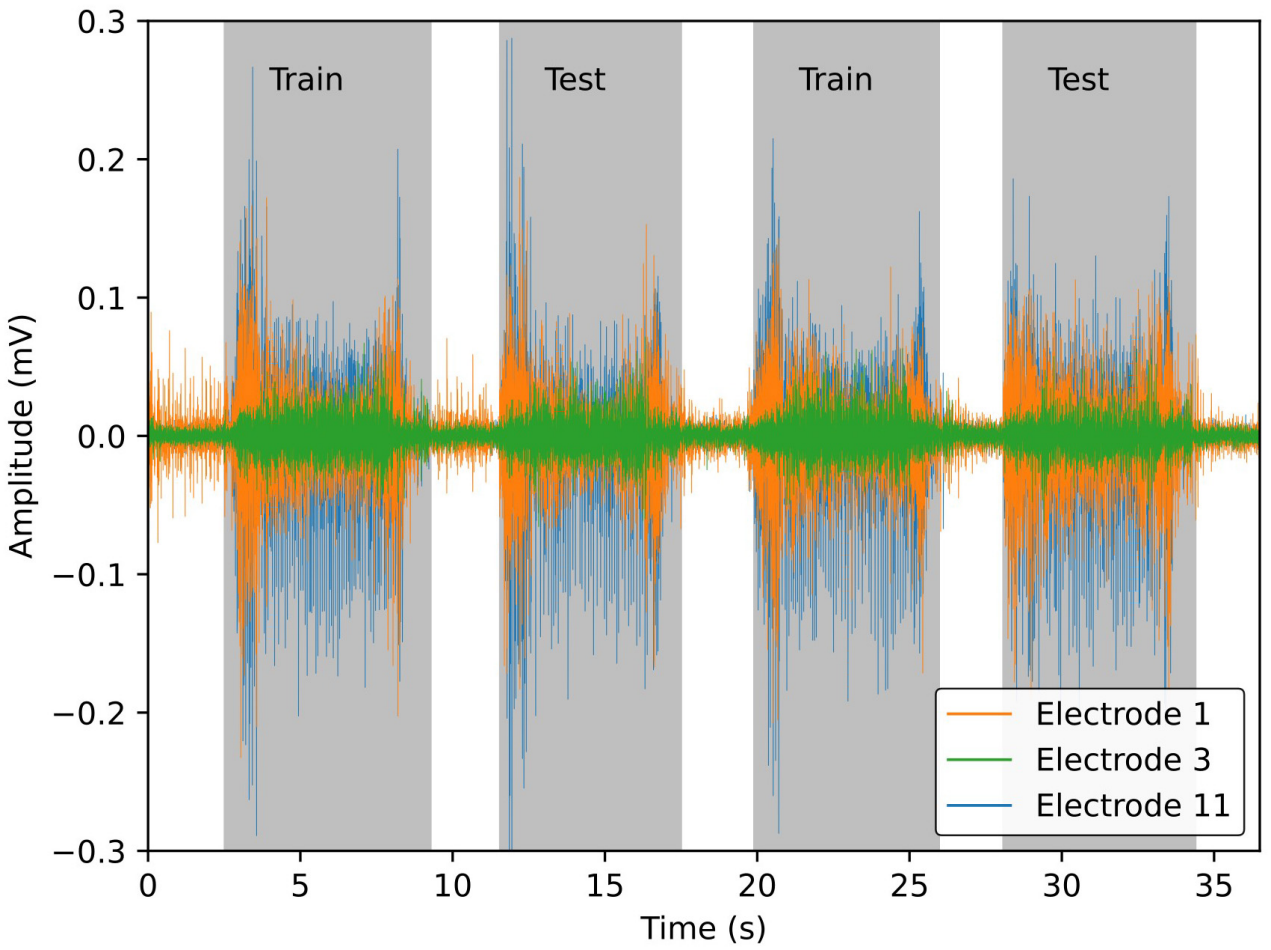
Example sEMG signals from 3 of the 14 sensors, for visual clarity, from DB6 (Palermo et al., 2017). Gray bounding boxes differentiate periods of muscle activity from rest. Odd repetitions of muscle activation are stored in the training set and even repetitions in the testing set. The windows from the rest periods are randomly undersampled from the entire signal to ensure a balanced dataset.

As per the original DB6 analysis, each session was segmented into a training and testing set—the odd repetitions used for training and the even for testing, example shown in Figure 2. This provided a roughly 50:50 split of the dataset and ensured that there was no information leakage between the two sets. Due to having a rest period between each activation, a class imbalance exists favouring the rest class. As such, the rest windows were undersampled such that no class was significantly biased in training or testing. The average number of windows of the activity classes was used as a target for the undersampling. All pre-processing was performed using MATLAB R2023a (MathWorks).

### 2.3 Dataset selection paradigms

Three dataset selection paradigms were considered and evaluated, confidence retraining, Edited Nearest Neighbour, and a novel SNR-based approach.

#### 2.3.1 Confidence retraining

Confidence-based retraining (CR) uses the classifier's largest computed class probability for each window to decide whether it is kept for retraining (Sensinger et al., 2009). For most classifiers, a probability vector is output and the predicted class is the index with the maximum probability. A window is retained for retraining if its maximum probability is equal to or above the threshold. Previously, Sensinger et al. (2009) compared supervised and unsupervised confidence-based retraining and found supervised to perform best. As such, the supervised method was employed in this work, where the retraining class for each accepted window uses its true label, rather than the predicted output of the previous classifier.

A percentage was selected to ensure that the most probable class was significantly more probable than any other class (i.e., the sum of all 7 other class probabilities is <= 100−*CR*_*threshold*_%). Conversely, a threshold too high could lead the paradigm to retain too few windows, which could impact performance. A previous study by Robertson et al. (2019) recommended that the confidence thresholds for rejection be between 60 and 75% to permit responsive controllers. To meet these three conditions, a 75% threshold was used in this work.

#### 2.3.2 Edited nearest neighbour

Edited Nearest Neighbour (ENN) is implemented using an adjusted version of the algorithm by Ding et al. (2019), (See Algorithm 1). First, the training data are applied to a *k*-Nearest Neighbour tree. Each window in the new training dataset is assessed against its 7 nearest neighbours using the previously extracted time-domain features. The nearest neighbour tree is created using the KDTree object from the scikit-learn Python library (Pedregosa et al., 2011). The inverse distance from each neighbour is used as a weighting for which class the window belongs to. If the class with the maximum weighting matches the true class assigned to the window, it is selected for retraining. Due to the 10 ms step of the 200 ms sliding window, the neighbouring window will contain 190 ms (95%) of the same data as the previous window. Thus, neighbours with high similarity in information must be ignored, or else the ENN paradigm will retain all windows. Therefore, the nearest 20 neighbours are excluded when considering a window for retraining. This should typically exclude the 10 neighbours on either side of a window, which contain 50% or more overlapping information.

**Algorithm 1:** ENN-based retraining.

Create a KDTree from the new dataset, *X*
for window, *w*, in *X* **do**
Get nearest neighbours 21 through 27, *N*
Create a zero vector of length 8, *V*_0..7_
for neighbour, *n*, in *N* **do**
Calculate distance, *d*, between *w* and *n*
Add $\frac{1}{d}$ to *V*_*i*_ where *i* is the class of *n*
end **for**
Expected Class = argmax(*V*)
if Expected Class = Known Class of *w* **then**
Accept *w* into retraining set
end **if**
end **for**

#### 2.3.3 Quality acceptance

Unlike CR and ENN which select windows for retraining based on machine learning algorithms, the QA paradigm proposed here relies solely on a single feature, the SNR. SNR is a quality metric; it is the ratio of the power of the sEMG during volitional muscle activation vs. rest. Low SNR can indicate an electrically noisy environment, poor electrode contact, or electrode movement. The paradigm seeks to provide a simplistic method of assessing a singular window's suitability for retraining, based on a general prior conception of quality. In comparison, the evaluation performed by the CR and ENN is computationally more complex and relies specifically on signals that have been processed by the existing system, which could skew their performance.

The SNR of a window was calculated using the equation in Table 1. A direct thresholding approach is used to select windows. A threshold SNR of >1.8 dB is based on previous work (Chang et al., 2020). As the recording electrodes are placed evenly around the arm, not all will detect a signal during muscle activation. Therefore, only the three largest SNRs for each window are compared with the threshold.

These three paradigms allow comparison of machine learning-based methods, the use of a secondary deterministic machine learning assessor, and a purely data-based paradigm.

### 2.4 Classifiers

**Figure 3:**
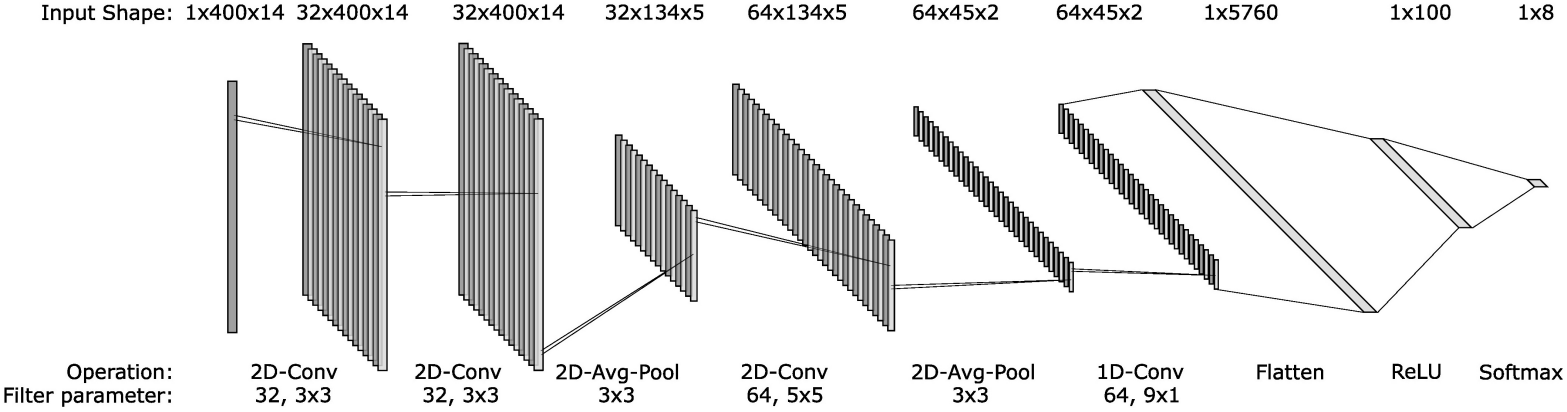
Architecture of the convolutional neural network used to classify sEMG signals. The input vector of each layer is presented above the structure, whilst the operations at each layer and any parameters are given below. All layers used a ReLU activation except the output layer, where a softmax activation was used. The architecture is based on the one presented by Atzori et al. (2016).

Classification performance was assessed using an LDA implemented with scikit-learn (Pedregosa et al., 2011) and a CNN implemented using Tensorflow (Abadi et al., 2015). The CNN structure (Figure 3), was as proposed by Atzori et al. for sEMG classification and used an Adam optimiser with a learning rate of 0.001, reduced to 0.0001 for retraining (Atzori et al., 2016). LDA and CNN are common classifiers in sEMG literature and were selected to confirm that the results are consistent between traditional and deep learning approaches (Khan et al., 2020). In addition, Sensinger et al. (2009) suggested that neural network-based models may be inherently more suitable for retraining as they can be readily adjusted with new data. As such, a comparison of the two classifier's performances using the retraining paradigms is presented. As a traditional machine learning algorithm, the LDA was trained and tested using the extracted time domain features. The CNN, however, used the signal data from the window as the convolution layers learn their own feature mapping during training. All models were trained and tested on a PC containing an Intel i9-11900 and an NVIDIA RTX 3080.

### 2.5 Dataset augmentation

The purpose of retraining the classifier is to adapt to the non-stationarity of the sEMG. Whilst DB6 encapsulates some of this variability, it is, in general, a high-quality dataset. As such, it was artificially augmented to degrade the overall quality (Chang et al., 2020). An augmented dataset (AB6) was constructed by adding pink noise to the original data. The pink noise for each recording was scaled by the greatest integer value which did not bring the average SNR below the 1.8 dB threshold. This ensured that the added noise did not saturate the original signals or cause paradigms to remove too many windows. The mean and standard deviation of the three largest SNRs were 5.53 dB ± 3.58 and 2.44 dB ± 1.91 for DB6 and AB6, respectively. Pink noise was used because its magnitude is scaled inversely to its frequency. Therefore, it predominantly affects the lower frequency range of the sEMG (<500 Hz) whilst adding some high-frequency noise. AB6 was then segmented according to the windowing and pre-processing of the base dataset.

### 2.6 Paradigm comparison

**Figure 4:**
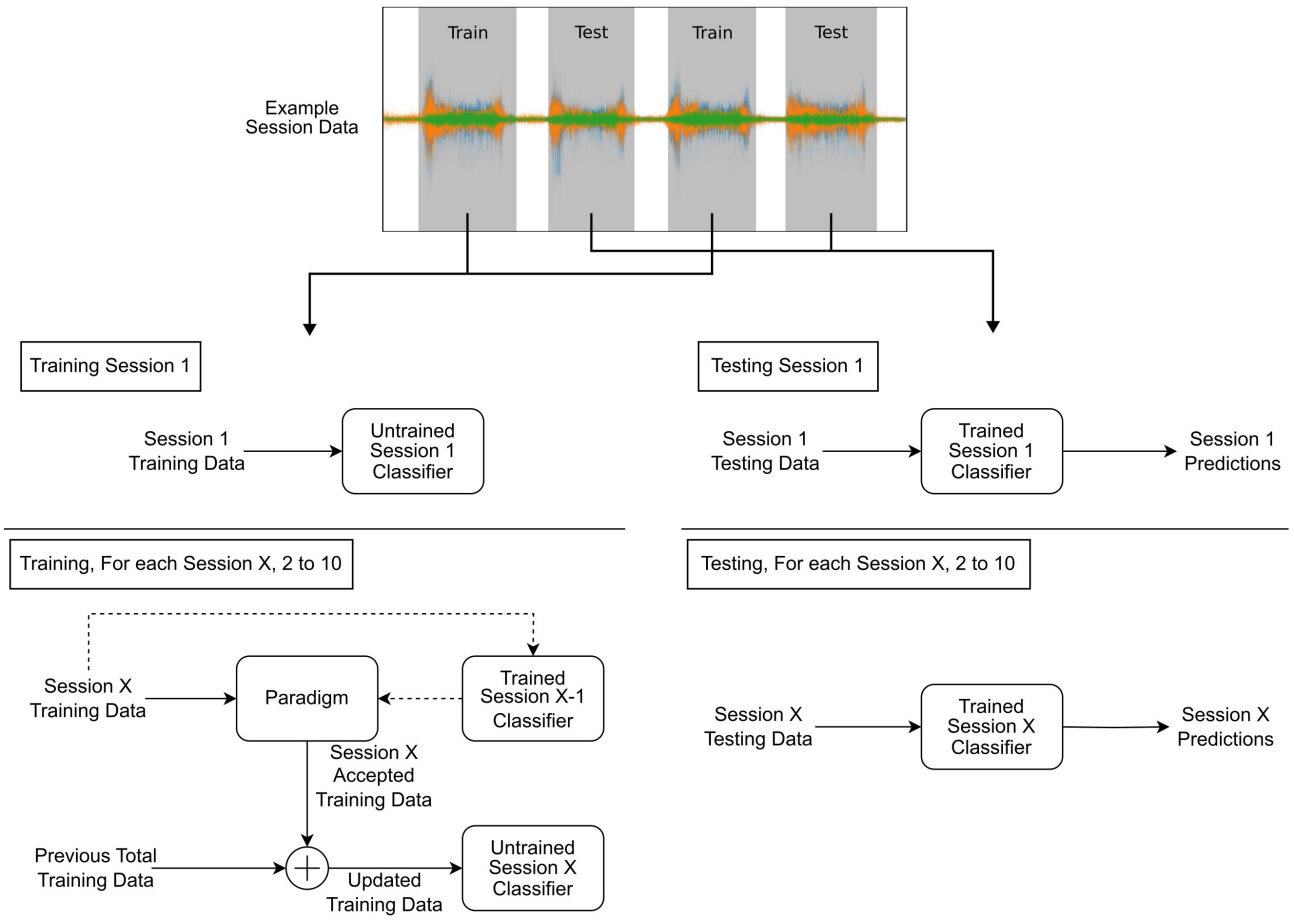
Diagram depicting the training and testing processes. Example session data are shown split into training and testing, with training procedures on the right block diagram and testing on the left. Each diagram is split showing the initial session 1 procedure and the subsequent paradigm-based retraining process for the remaining sessions.

The process of training and testing the classifiers using the data selection paradigms is presented in Figure 4. First, the base classifiers (LDA and CNN) were trained using the training set of session 1, and baseline performance was established using the corresponding test set. The training set of session 2 was then exposed to the paradigm, and the accepted windows were used to retrain the classifier. Next, the test set from session 2 was used to establish the performance of the retrained classifier. This process was then repeated for the remaining sessions.

Scheme et al. (2013) previously showed that the rejection of low-confidence windows can significantly improve the overall classifier accuracy. In an online system, rejection is the equivalent of not changing the current motor outputs for that window. As long as the rejection rate is not so great that updates to the output become too infrequent, this can permit more controllable pattern recognition systems. Therefore, the three paradigms were also used to reject windows from the test set. In this case, only testing windows accepted by the paradigms were considered for the performance metrics.

In addition to being compared with one another for both retraining and rejection, each paradigm is compared individually with an existing post-processing technique for overlapping windows, majority vote. The majority vote aims to smooth classifier output by evaluating neighbouring window outputs for each update step and selecting the most common class from these. This has been shown to improve accuracy compared to classification alone (Tigrini et al., 2024). The majority vote can be applied as long as the overlap is smaller than the acceptable system delay, 100 ms (Farrell, 2011). Equation 1 provides the limit for the value of *m* neighbours that can be used from either side of a window to meet the acceptable delay requirement; thus, the number of neighbours equals 2*m*+1 (Englehart and Hudgins, 2003). Given the 10 ms overlap used, 21 neighbours were used to assess the majority vote. Comparison of the majority vote with the paradigms was performed following retraining by applying either majority vote or paradigm-based rejection to each of the sessions test scores.


(1) $$\begin{array}{l} m\times \text{Overlap}\leq 100ms \end{array}$$

The primary performance indicator was classification accuracy, calculated as the percentage of correct classifications from the total on each testing set. Confusion matrices comparing the predicted classes with the true classes and the change in F1-score across sessions were then used to analyse the paradigm's impact on the individual class accuracy.

The Wilcoxon signed-rank test was used to determine statistically significant differences in classifier accuracy for all participants and sessions, as recommended by (Demšar 2006). The output accuracies for each combination of classifier and paradigm were assessed against their paired output accuracies of the same classifier where no retraining was undertaken. An alpha value of 0.05 is used for all tests of significance.

## 3 Results

### 3.1 Baseline

**Figure 5:**
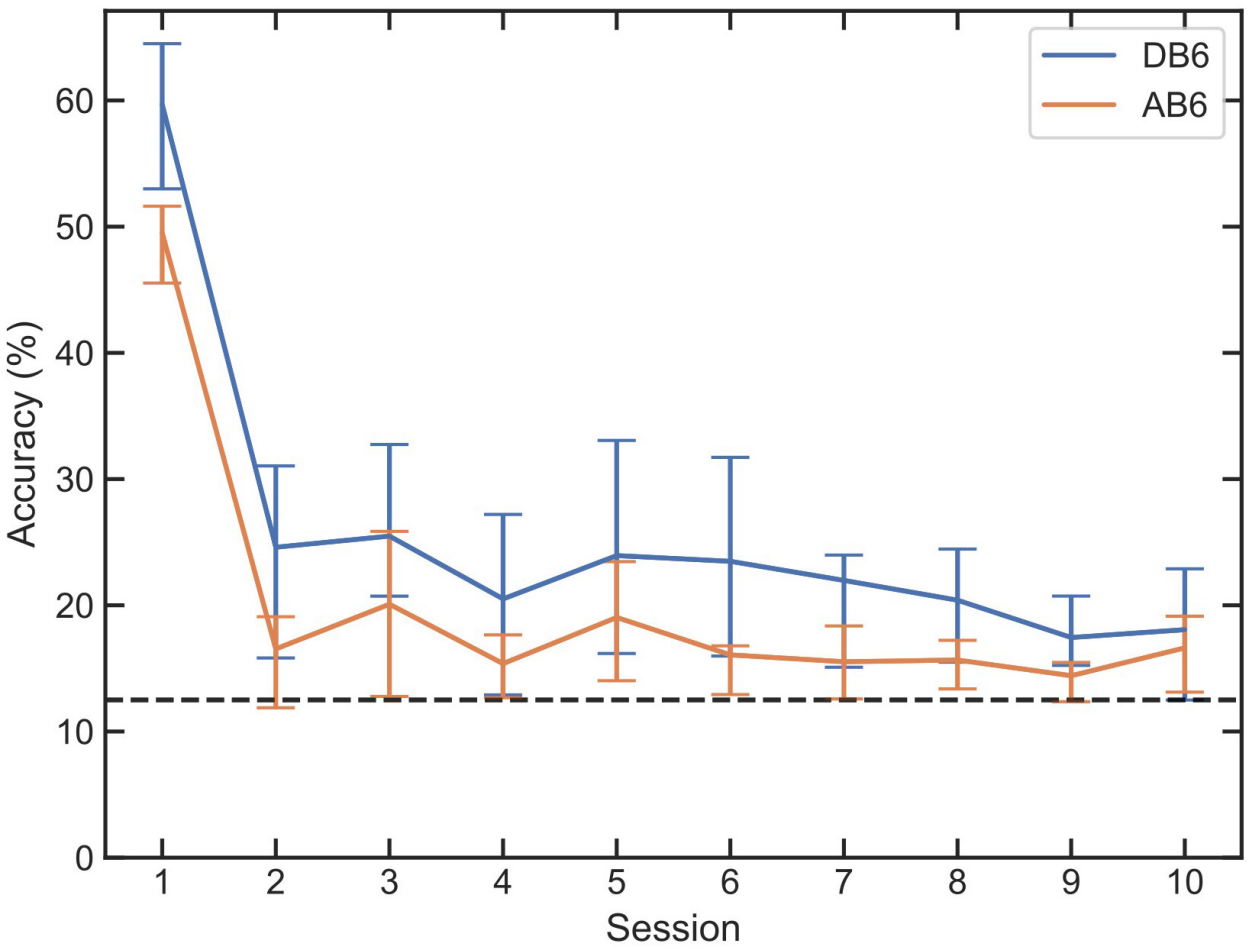
Mean accuracy and interquartile range of LDAs trained on session 1 of each participant, for both DB6 and AB6. Every session is then tested with the LDA with no retraining intervention, to establish a baseline of subsequent session accuracy. A dashed line represents the 12.5% random chance of an 8-class classification.

Figure 5 presents the mean accuracy of all LDA classifiers for each of the 10 participants trained on session 1 and tested on all 10 sessions of each dataset without retraining (baseline accuracy). Accuracy drops significantly between sessions 1 and 2, with an immediate mean accuracy loss across all participants and both datasets of 35.37%. An overall mean loss between sessions 1 and 10 of 41.94% is recorded. For some participants, accuracy drops below chance (12.5%) as shown in black in Figure 5.

### 3.2 Retraining

**Figure 6:**
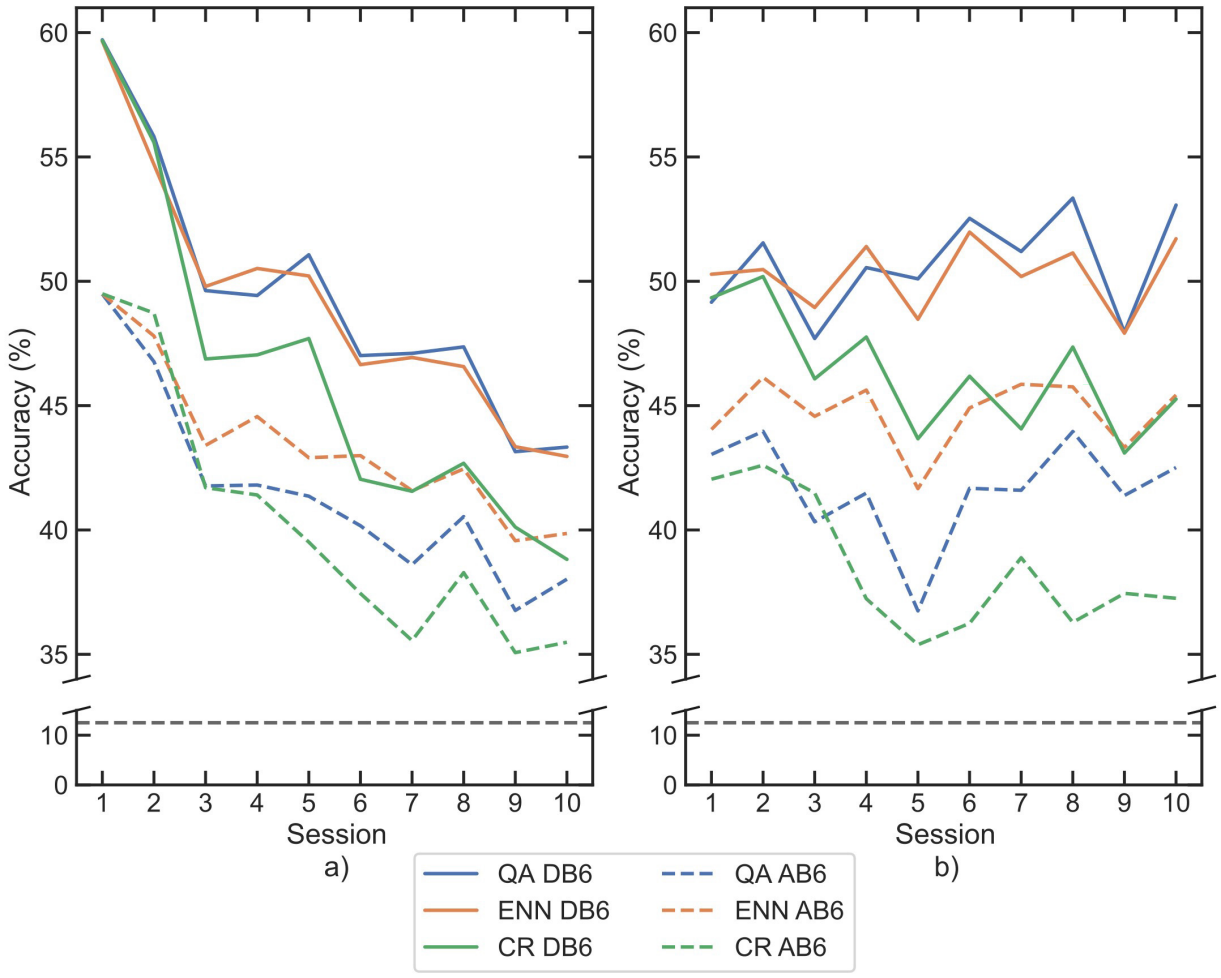
Mean classification accuracies for participants across each session following retraining by each paradigm, for each classifier **(a)** LDA, **(b)** CNN. Paradigms are indicated by line color, whilst datasets are separated between solid and dashed lines. The 8-class chance line is shown in black dashes.

The median accuracy for each session is shown for both LDA and CNN classifiers on both datasets in Figure 6. All 3 retraining paradigms significantly improve the accuracy (*p*-values <0.001) between each session compared with the baseline for all classifier and dataset combinations. In the final session (session 10), accuracies increased against baseline by a mean of 26.89% for QA, 21.86% for CR, and 27.65% for ENN across both classifiers and datasets. The CR paradigm on AB6 presents the minimum improvement in session 10 accuracy, with an increase of 18.86%. The QA and ENN paradigms both improved accuracy significantly compared to the CR paradigm (*p*-value <0.001).

Across all 4 combinations of classifier and dataset, the ENN paradigm performed best at improving inter-session accuracy, with a mean loss between sessions 1 and 10 of 5.88% accuracy. QA was second with a mean loss of 6.12%, and CR was last with a mean loss of 10.93%. The accuracy loss was more significant with LDA than with CNN, but the paradigm performance trend persists in each classifier.

### 3.3 Class imbalance

A common issue in classification scenarios is the performance impact resulting from imbalanced data, where a significantly over-represented class within the dataset skews the classifier output (Kumar et al., 2021). In a context where the dominant class represents very similar information, such as the rest class in the DB6, undersampling the class can resolve the imbalance. However, in the retraining paradigms presented here, the new data are being selected based on the paradigms' conditions, which therefore could result in an imbalance in data. In some cases, this imbalance could be further reinforced in subsequent sessions. Therefore, assessing the paradigms' effect on class balance in both the selection of windows for retraining and the classification performance must be considered.

**Figure 7:**
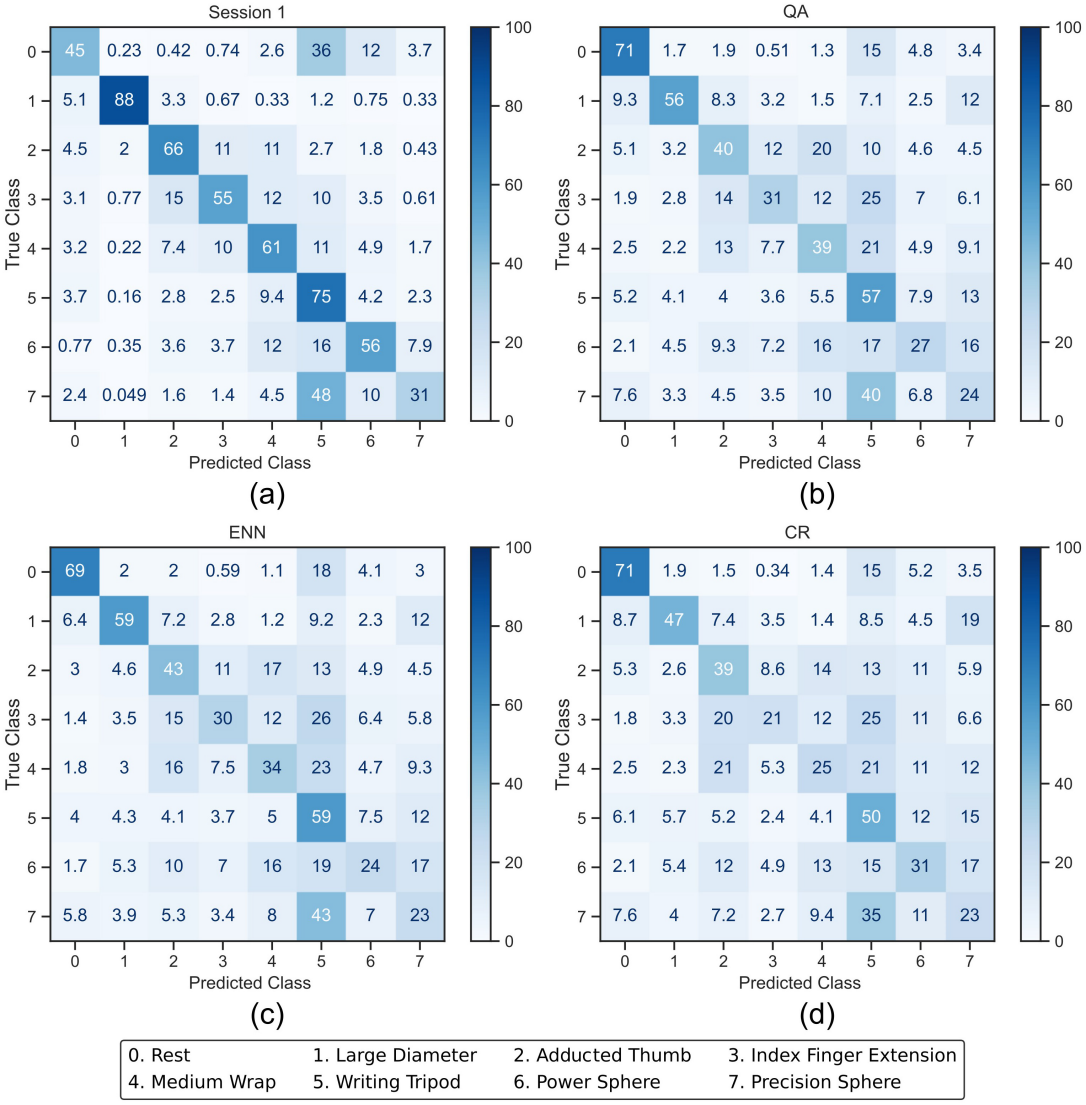
Averaged confusion matrices (in %) from the LDA classifier outputs. **(a)** shows the session 1 output of the LDA after being trained with the full training set. **(b–d)** show the session 10 output after the LDA was retrained using QA, ENN, and CR, respectively.

**Figure 8:**
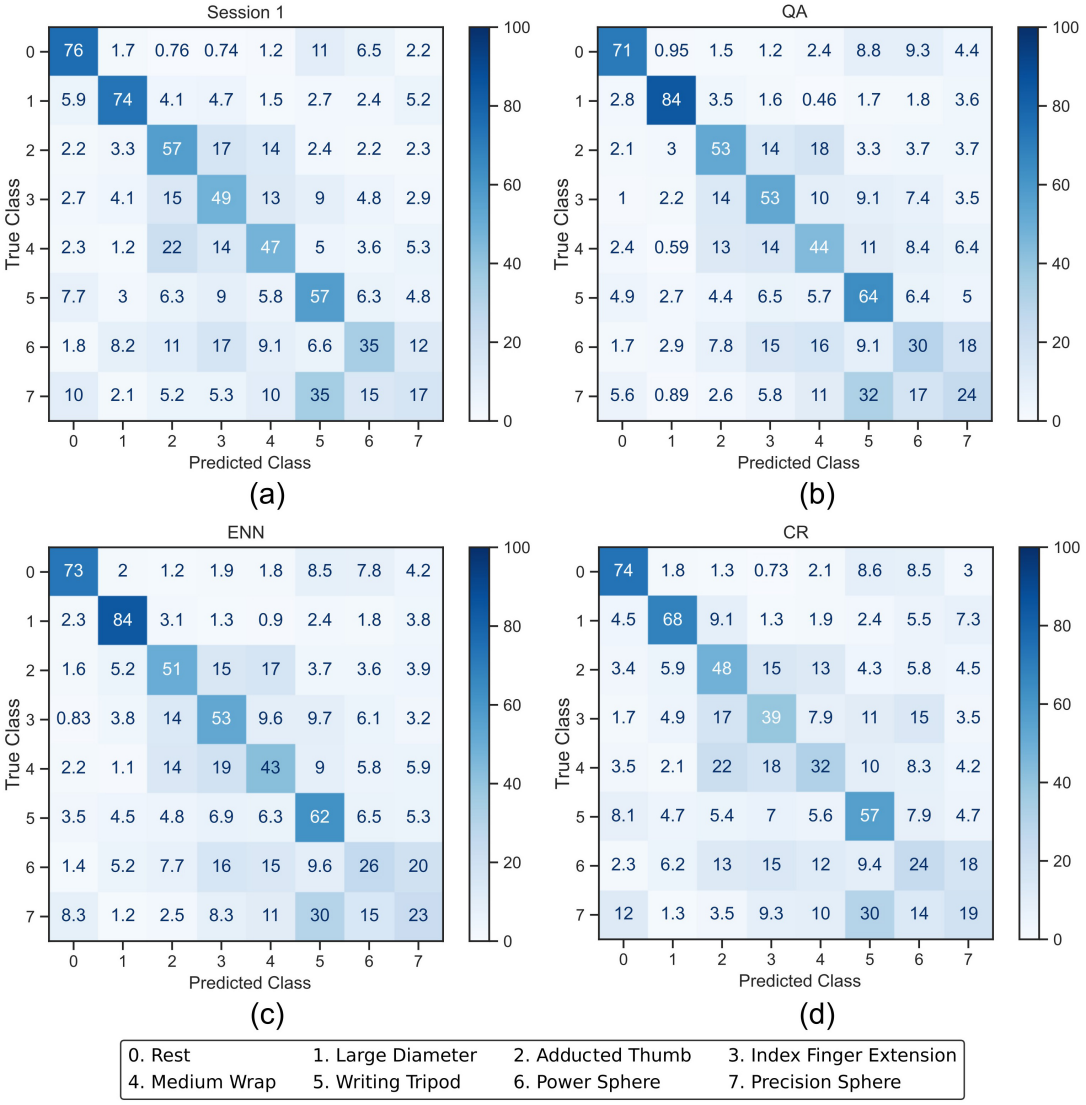
Averaged confusion matrices (in %) from the CNN classifier outputs. **(a)** shows the session 1 output of the CNN after being trained with the full training set. **(b–d)** show the session 10 output after the CNN was retrained using QA, ENN, and CR, respectively.

To investigate the impact on class balance, averaged confusion matrices and the F1-score were used. The confusion matrices illustrate the actual accuracy of each class as a percentage along the diagonal, whilst the F1-score change between sessions presents a summary of the degree by which each class's performance changes. Figures 7, 8 present the averaged confusion matrices from all participants' LDA and CNN classifier outputs, respectively. Subfigure a) shows the classifier trained on Session 1, whilst subfigures b), c), and d) show the classifier post-retraining with each paradigm and testing on Session 10. When using LDA, the accuracies of the activation classes (1–7) decrease on all paradigms between 7 and 41%, whilst the rest class accuracy starts low at 45% and increases by 24–26%. This improved accuracy of the rest class was expected; rest should be relatively consistent across all sessions due to minimal muscle activity, and new exemplars that readily resemble noise during the recording session are added to the training pool. However, these results suggest the LDA performance was greater than the baseline due to improved rest accuracy at the detriment of activation accuracy, presenting imbalanced retraining.

The CNN classification accuracy of the rest class starts higher at 76% and decreases slightly for each paradigm by a maximum of 5%. The individual activation class accuracy change occurs between a decrease of 15% and an increase of 10%. This presents a more balanced retraining than LDA and reflects the overall accuracy trend in Figure 6.

In the case of the LDA using the CR paradigm, the classes with the lower initial accuracy (3, 4, and 7) experience a further drop in accuracy. This indicates that less new windows for these classes were added in the retraining process because the initial classification rate was lower. This effect was less apparent on the CNN, where the accuracy of classes 6 and 7 increases.

Table 2 shows the change in F1-score between session 1 and session 10. A negative change represents a lower score in session 10 and thus shows that the class's overall precision and recall have reduced. For the retraining to have a balanced impact, it would be expected that the precision and recall would change by a zero or small non-zero amount. For LDA, the F1 score of the rest class increased on average by 0.082 compared to the significant decrease of all activation classes with a range of -0.018 to -0.335.

**Table 2:** Median and IQR F1-score differences for each class and Macro score following retraining between Session 1 and Session 10 for each classifier and paradigm combination.

**Classifier**	**Paradigm**	**Median F1-score difference between session 1 and 10, per class**								
		**0**	**1**	**2**	**3**	**4**	**5**	**6**	**7**	**Macro**
LDA	QA	0.094(0.000 to 0.174)	-0.234(-0.373 to -0.081)	-0.245(-0.331 to -0.080)	-0.174(-0.342 to -0.042)	-0.170(-0.277 to -0.158)	-0.088(-0.135 to -0.033)	-0.100(-0.278 to -0.032)	-0.014(-0.029 to 0.006)	-0.140(-0.158 to -0.062)
	ENN	0.087(-0.055 to 0.134)	-0.230(-0.329 to -0.038)	-0.192(-0.223 to -0.045)	-0.155(-0.332 to -0.021)	-0.182(-0.246 to -0.154)	-0.079(-0.138 to -0.016)	-0.093(-0.280 to 0.010)	-0.018(-0.036 to 0.009)	-0.128(-0.132 to -0.042)
	CR	0.066(-0.074 to 0.123)	-0.335(-0.524 to -0.076)	-0.239(-0.432 to -0.155)	-0.217(-0.418 to -0.163)	-0.276(-0.370 to -0.207)	-0.105(-0.252 to -0.083)	-0.089(-0.313 to 0.055)	-0.029(-0.055 to -0.007)	-0.179(-0.237 to -0.103)
CNN	QA	-0.027(-0.082 to 0.124)	0.107(0.069 to 0.171)	0.028(-0.033 to 0.105)	0.016(-0.030 to 0.100)	-0.008(-0.128 to 0.045)	0.018(-0.045 to 0.083)	-0.015(-0.193 to 0.041)	0.033(-0.030 to 0.088)	0.016(-0.021 to 0.052)
	ENN	-0.042(-0.075 to 0.098)	0.058(0.013 to 0.100)	0.046(-0.062 to 0.129)	0.017(-0.019 to 0.092)	-0.048(-0.150 to 0.038)	-0.001(-0.097 to 0.131)	-0.097(-0.133 to 0.043)	-0.005(-0.086 to 0.161)	-0.012(-0.021 to 0.056)
	CR	-0.034(-0.144 to 0.095)	0.011(-0.215 to 0.134)	0.027(-0.048 to 0.060)	-0.018(-0.074 to 0.025)	-0.104(-0.310 to 0.058)	-0.017(-0.058 to 0.094)	-0.057(-0.150 to 0.018)	-0.053(-0.127 to 0.002)	-0.005(-0.091 to 0.011)

For CNN, the F1 score shows a more random distribution of increases and decreases with activation scores changing between -0.104 and 0.107. This again indicates that the performance of the retraining was not at the significant detriment of any particular class. The negative change in the rest class reflects the accuracy loss in Figure 8. The interquartile ranges (IQRs) of the CNN also demonstrate a tighter change in score than the LDA. From the Macro F1-scores, it can be seen that only the QA paradigm has a positive overall change on average between the participants of 0.016. Whilst the ENN paradigm also has a positive accuracy change in Figure 6, the negative F1-score of -0.012 indicates that the misclassification rate increases slightly.

### 3.4 Window retention

The retention rate of the paradigms was also compared. Table 3 presents the mean retention of each class across the participants for all paradigm, classifier, and dataset combinations. The QA and the ENN paradigm retention percentages are the same for each classifier as the paradigms are deterministic and the same features (SNR and time-domain, respectively) are input for either classifier. The QA paradigm retains 7% of the rest class in AB6 and 45% in DB6, as the rest class should have no activity this retention rate was expected. On DB6, almost all muscle activity windows are retained, and on AB6, a mean of 83% of all activity windows are retained. It can be seen that class 5 was significantly affected by the augmentation.

**Table 3:** Mean percentage class retention of the training set of all classifiers, dataset, and paradigm combinations calculated across all participants.

**Classifier and dataset**	**Paradigm**	**Mean class retention (%)**								**Mean retention (%)**
		**0**	**1**	**2**	**3**	**4**	**5**	**6**	**7**	
LDA-DB6	QA	45.35	99.47	99.20	99.43	99.44	94.95	99.40	83.37	90.08
	ENN	82.85	68.41	50.67	56.17	56.91	60.42	70.28	57.31	62.88
	CR	84.98	72.15	42.70	53.74	47.40	59.52	70.95	57.19	61.08
LDA-AB6	QA	7.90	85.19	92.76	87.44	94.54	47.22	91.56	78.86	73.18
	ENN	74.24	73.00	54.59	63.68	61.41	55.27	73.12	64.24	64.94
	CR	55.72	58.71	36.78	43.57	40.44	36.74	69.10	58.89	49.99
CNN-DB6	QA	45.35	99.47	99.20	99.43	99.44	94.95	99.40	83.37	90.08
	ENN	82.85	68.41	50.67	56.17	56.91	60.42	70.28	57.31	62.88
	CR	71.47	69.39	63.84	58.51	61.27	60.02	69.57	62.71	64.60
CNN-AB6	QA	7.90	85.19	92.76	87.44	94.54	47.22	91.56	78.86	73.18
	ENN	74.24	73.00	54.59	63.68	61.41	55.27	73.12	64.24	64.94
	CR	81.81	72.75	65.37	63.32	65.16	72.78	73.51	63.82	69.81

Summary means allowing an overview comparison of the entire retention of a combination given at the end of each row.

The ENN and CR paradigms achieved improved accuracies using significantly less of the dataset. ENN retained 63% of the windows in both DB6 and AB6; however, the individual class percentages vary, further demonstrating the impact of the augmentation. The balance of class retention was typically weighted toward the rest class for both paradigms. This was reduced by ~ 10% on AB6 because the augmentation caused the rest to become less distinct from muscle activity. The CR paradigm retained less than the ENN paradigm with the LDA by 1.80% and 14.50% on DB6 and AB6, respectively, and more with the CNN by 1.71% and 4.96%, respectively.

### 3.5 Paradigm informed window rejection

**Figure 9:**
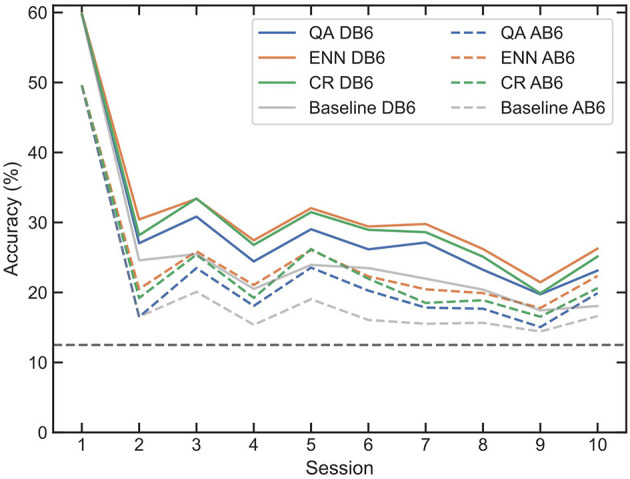
Mean accuracy of LDAs trained on session 1 of each participant. Subsequent trials are tested with the LDA using the paradigms to reject windows which failed to meet their acceptance criteria. Gray lines represent the DB6 and AB6 baselines.

The rejection of windows and the intersession performance were assessed across all three paradigms. A single instance of each classifier was trained on session 1 per participant, and the classifiers were not re-trained on subsequent sessions. For the CR and QA paradigms, windows that fell below the retraining acceptance threshold were rejected from testing. Similarly, for ENN, where the paradigm suggested a class different from the true label, the window was rejected. Compared to baseline, small improvements were observed (Figure 9); however, the general improvement was worse than using the paradigms for retraining. On DB6, the immediate mean loss for QA was 32.78%, ENN was 29.47%, and CR was 31.63%. The overall mean loss between sessions 1 and 10 for QA was 36.69%, ENN was 33.63%, and CR was 34.67%. Positively, however, the mean loss follows a very similar pattern for each dataset to that of the baseline. This indicates that the rejected windows are outliers that would have led to incorrect predictions.

The Wilcoxon test reported that all results were statistically different from both the baseline and their paired-equivalent retraining results (*p*-value <0.001).

### 3.6 Combining retraining with rejection

**Figure 10:**
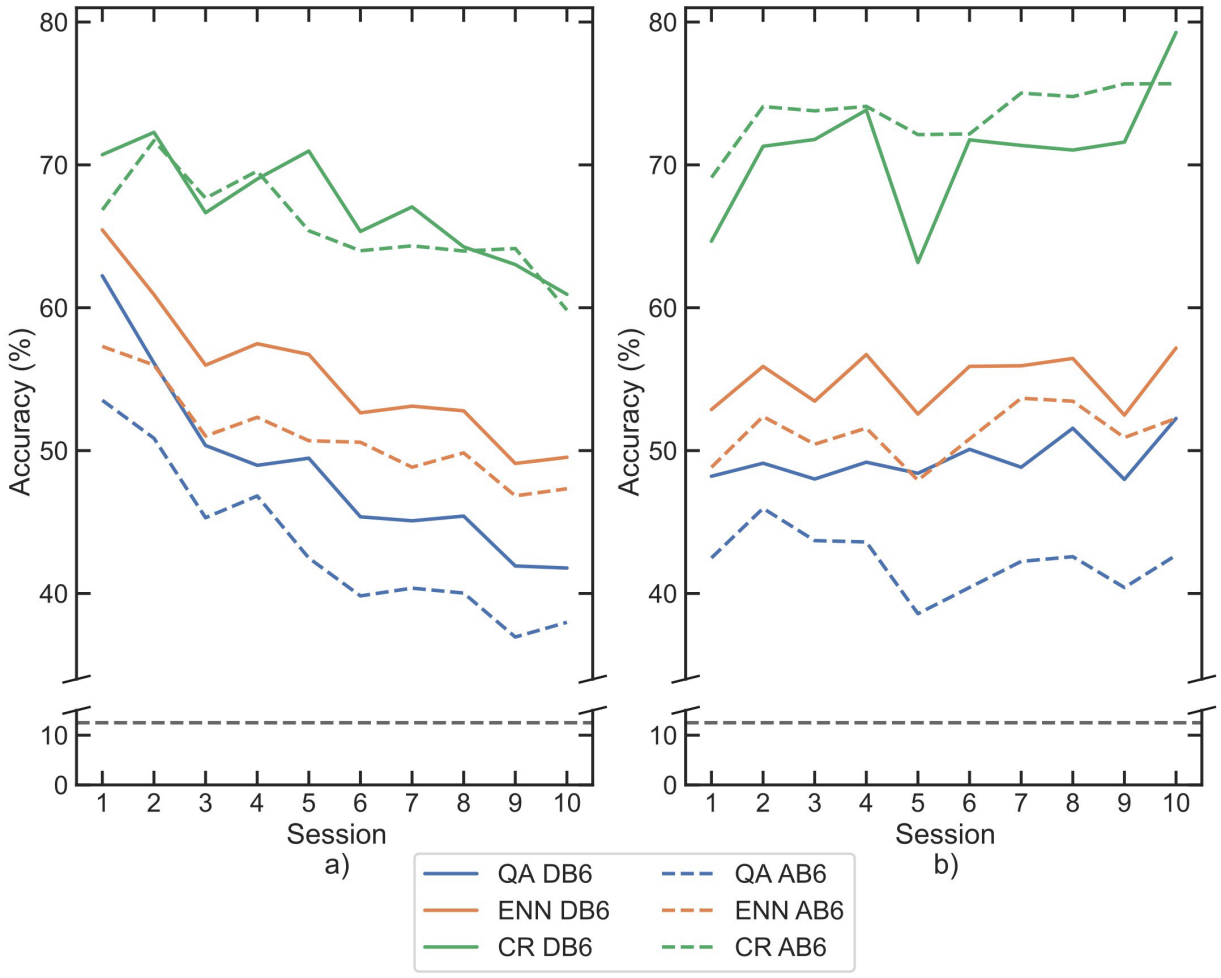
Mean classification accuracies for participants across each session following retraining and subsequent rejection by each paradigm, for each classifier **(a)** LDA, **(b)** CNN. Paradigms are indicated by line color, whilst datasets are separated between solid and dashed lines. The 8-class chance line is shown in black dashes.

Although rejection alone did not improve classification accuracy between sessions, it should still further improve the retraining of a classifier. Prior work has shown the efficacy of rejection in improving a classifier's performance on testing data from the same recording session. As such, if the rejection is applied after the retraining, then a similar improvement should be observed. Figure 10 shows the mean accuracy of the 10 participants when each paradigm was used both for window selection in the training set and for rejection in the test set.

The classification accuracy of CR increased across all sessions, with final scores greater than DB6 baseline by 42.87% with the LDA and 61.20% with the CNN, confirming its efficacy for rejection as per Scheme et al. (2013). The combined rejection-retrain accuracies were then compared to retraining only. Table 4 shows the mean percentage of the test dataset that was rejected by each paradigm. The same Wilcoxon signed-rank test was used to compare the paired data. Both the CR and ENN paradigms had a significant difference in accuracy for all combinations of dataset and classifier (*p*-value <0.001 ). The CR paradigm's mean session 1 accuracy improved by 13.18% on DB6 and by 22.21% on AB6, whilst session 10 accuracy improved by 28.06% on DB6 and by 31.39% on AB6.

**Table 4:** Mean percentage rejection rate of windows from the testing set for each classifier, dataset, and paradigm combination, across all participants and sessions.

**Classifier-dataset**	**Paradigm**	**Mean rejection (%)**
LDA-DB6	QA	10.89
	ENN	32.72
	CR	61.06
LDA-AB6	QA	33.17
	ENN	39.15
	CR	73.17
CNN-DB6	QA	10.89
	ENN	32.72
	CR	65.91
CNN-AB6	QA	33.17
	ENN	39.15
	CR	79.33

The accuracy of the ENN paradigm increased less so, in session 1 by 4.19% on DB6 and by 6.29% on AB6, and in session 10 by 6.02% on DB6 and 7.14% on AB6. The percentage of rejection of AB6 for all paradigms was higher than that of DB6. This was reflected in the accuracy improvement on AB6 for the CR and ENN paradigms.

For the QA paradigm, accuracies on DB6 for both classifiers were found to be significantly different (*p*-value <0.001); however, on AB6, both classifiers were not significantly different (*p*-value: LDA = 0.16, CNN = 0.39). From Table 4, it can be seen that the QA paradigm rejected a smaller portion of the windows. However, the QA paradigm retains <10% of the rest class (0) on AB6 (Table 3). This suggests why accuracy does not improve significantly with rejection as the paradigm rejects a readily classifiable class. The F1-score difference between sessions 1 and 10 was calculated again and presented in Table 5. As with solely retraining, the LDA showed a significant improvement in performance for the rest class with an average increase of 0.267 and a decrease for all activity classes ranging between -0.108 and -0.426. The larger respective increase and decrease further reinforce the fact that the LDA develops a bias toward the rest class during the retraining process. The CR paradigm has the greatest Macro decrease over the 10 sessions of -0.277, whilst its accuracy was the highest of the paradigms. This value results from the high misclassification rate of classes 2, 3, and 4 observed both from their low F1-scores and the high rejection rate of the paradigm. The F1-score changes of the CNN are again much more balanced than that of the LDA with a distribution of positive and negative changes amongst all the classes. The ENN paradigm achieves the largest Macro F1-score change in the positive direction across both classifiers.

**Table 5:** Median and IQR F1-score differences for each class and Macro score following retraining and rejection between Session 1 and Session 10 for each classifier and paradigm combination.

**Classifier**	**Paradigm**	**Median F1-score difference between session 1 and 10, per class**								
		**0**	**1**	**2**	**3**	**4**	**5**	**6**	**7**	**Macro**
LDA	QA	0.235(0.050 to 0.424)	-0.339(-0.414 to -0.127)	-0.296(-0.379 to -0.164)	-0.207(-0.348 to -0.112)	-0.199(-0.263 to -0.183)	-0.091(-0.147 to -0.028)	-0.269(-0.460 to -0.201)	-0.154(-0.282 to 0.006)	-0.182(-0.235 to -0.103)
	ENN	0.264(0.026 to 0.378)	-0.276(-0.417 to -0.106)	-0.283(-0.373 to -0.133)	-0.203(-0.345 to -0.102)	-0.254(-0.316 to -0.213)	-0.108(-0.159 to 0.004)	-0.319(-0.399 to -0.181)	-0.112(-0.285 to 0.008)	-0.162(-0.238 to -0.085)
	CR	0.302 (0.069 to 0.400)	-0.253 (-0.909 to -0.045)	-0.382 (-0.652 to 0.010)	-0.426 (-0.526 to -0.216)	-0.436 (-0.680 to -0.296)	-0.155 (-0.257 to -0.066)	-0.234 (-0.582 to -0.083)	-0.210 (-0.315 to -0.021)	-0.277 (-0.365 to -0.177)
CNN	QA	-0.029(-0.122 to 0.243)	0.008(-0.067 to 0.109)	0.012(-0.031 to 0.040)	0.007(-0.053 to 0.084)	0.108(-0.044 to 0.265)	0.082(0.025 to 0.154)	-0.058(-0.084 to -0.032)	0.030(-0.004 to 0.100)	0.030(0.011 to 0.102)
	ENN	0.008(-0.063 to 0.274)	-0.007(-0.080 to 0.099)	0.034(-0.036 to 0.130)	0.045(-0.051 to 0.125)	0.213(-0.029 to 0.326)	0.050(0.011 to 0.136)	-0.033(-0.120 to 0.079)	0.014(-0.118 to 0.108)	0.038(0.005 to 0.096)
	CR	0.064(-0.054 to 0.235)	0.016(-0.014 to 0.078)	-0.013(-0.576 to 0.106)	-0.019(-0.105 to 0.077)	-0.145(-0.301 to -0.064)	0.062(-0.076 to 0.175)	-0.047(-0.241 to 0.035)	0.013(-0.181 to 0.186)	-0.019(-0.081 to 0.022)

**Figure 11:**
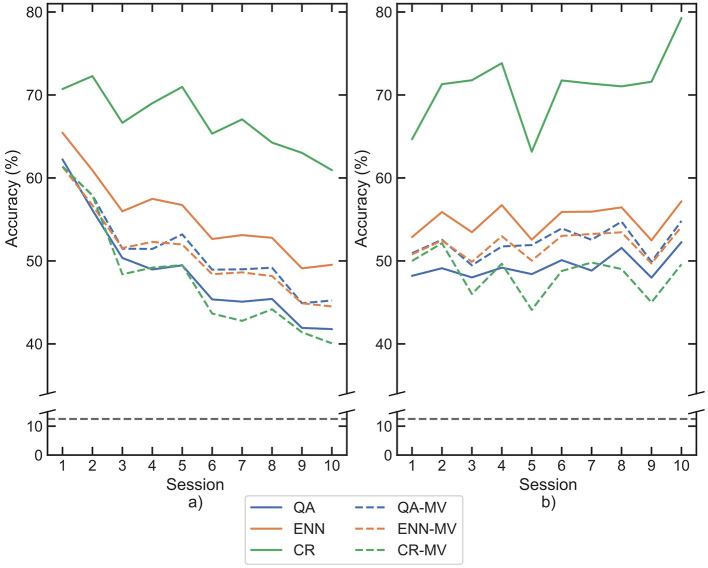
Mean classification accuracies for participants across each session following retraining by each paradigm and subsequent rejection or majority vote post-processing on DB6, for each classifier **(a)** LDA, **(b)** CNN. Paradigms are indicated by color, solid lines show paradigm based rejection, whilst dashed lines show majority vote results. The 8-class chance line is shown in black dashes.

The use of each paradigm for rejection after retraining was additionally compared with majority vote post-processing. The accuracy scores obtained on DB6 for both rejection-based and majority vote-based post-processing are provided in Figure 11. For both classifiers, the ENN and CR paradigms outperform the majority vote method. The ENN improved the overall mean of the dataset compared to the majority vote by 5.35% and 3.28%, whilst CR improved by 18.58% and 24.03% for the LDA and CNN, respectively. The QA paradigm performed worse than the majority vote method, scoring a 3.94% and 4.04% lower overall mean for each classifier, respectively. In all cases, differences were considered significant using the Wilcoxon test (*p*-values <0.001).

## 4 Discussion

This work has compared how three window selection paradigms impact the classification performance of sEMG data captured over 5 days in 10 separate recording sessions. The paradigms select windows of new data that should enable the respective classifier to adapt to non-stationarities and drift. The effect of the three paradigms was compared using both an LDA and a CNN to demonstrate their efficacy with traditional and deep machine learning classifiers. This work compares the direct performances of the paradigms, their effect on class balance, and their use in rejection and retraining, as well as in combination.

### 4.1 Classifier comparison

Although CNNs and LDA have been shown to achieve high classification accuracy (> 90%) on sEMG data (Pinzon-Arenas et al., 2019; Lorrain et al., 2011), there is limited work that compares their use in retraining. CNNs can be partially fit, allowing new training data to be introduced to the classifier at a later time, which more readily allows them to adapt to non-stationary inputs such as sEMG—as later training epochs influence the network's weights more. Alternatively, the LDA must be trained on all data at one time, which means that old data have as much influence on the classifier as new data. In this work, it has been shown that the initial accuracy in session 1 using the CNN is lower in both datasets than with the LDA by 10%. However, the mean reduction in accuracy for all paradigms is only 0.44% over the 10 sessions with the CNN and 14.83% with the LDA. The limited reduction in accuracy is evidence of the CNN's suitability for retraining. Its lower initial accuracy could be attributed to the general network structure, rather than fine-tuning the hyperparameters for each participant (Atzori et al., 2016).

In addition, the F1-scores presented in Tables 2, 5 show the CNN better preserves a balance of performance across all classes in both retraining and retraining with rejection. The LDA only shows positive F1-score changes for the rest class in both cases and a large negative average Macro change of -0.149 and -0.207, respectively. This indicates that its accuracy is achieved to the detriment of the activation classes. Alternatively, the CNN has a more balanced distribution of changes across all classes, further demonstrated by its smaller average Macro score on all paradigms of -0.0003 and 0.016, respectively. This reinforces the suggestion that the CNN classifier is more suited to retraining. These findings align with the proposal of Sensinger et al. (2009) that the iterative nature of the CNN may improve on the performance of an LDA in retraining.

### 4.2 Paradigm comparison

The importance of retraining is demonstrated in Figure 6; all three paradigms show a reduced loss of accuracy compared to the baseline loss by session 10 of 41.94% in Figure 5. When used for window selection retraining, both the QA and ENN paradigms outperform the CR paradigm, improving accuracy by ~5%. When considering the function of a prosthesis, even small improvements in accuracy can result in smoother and more intuitive control for amputees. Furthermore, an increase in accuracy suggests a more reliable pattern recognition scheme, indicating less frequent retraining may be necessary in subsequent sessions. In a clinical device, this could benefit amputees by reducing the time and cognitive load required to retrain the controller. In all cases, the use of the AB6 dataset reduces total accuracy across all sessions, with the mean starting accuracies for the LDA reduced by 10.19% and by 6.55% for the CNN.

From the retention data shown in Figure 2, the higher performance of QA is likely due to its high window retention rate. Although this could be a positive trade-off, high window retention rates could lead to storage issues on embedded devices if multiple sessions must be stored. Furthermore, over an extended period, overtraining could become present, resulting in accuracy reduction without systems to manage for this. The ENN and CR paradigms retain a significantly lower amount but still improve accuracy compared to the baseline; in the case of the ENN paradigm, a slight improvement is found over the QA paradigm (0.76%). The overall retention rates of the ENN and CR paradigms are similar in all combinations except LDA AB6 where the CR paradigm is lower by 10%. This indicates that the paradigm may require adjustable thresholds depending on the type of classifier used as the increased noise has a greater affect on the LDA. The impact of added noise is similarly observed with the QA paradigm as its retention rate is also reduced. As this paradigm does not depend on the classifier, if recordings were taken from an even noisier environment, the paradigm may fail altogether. However, this could also be used as a warning system in a prosthesis system for significantly poor quality recording.

As observed in Figure 7, the difference in accuracy between the ENN and CR paradigms is caused by their retention method. The CR paradigm retains classes on which the classifier predicts the answer confidently, so the loss of accuracy in classes 1, 2, and 5 is less than in the others. However, the ENN paradigm retains based on the similarities between windows before being introduced to the classifier, so its accuracy retention is more balanced, and its misclassification rates do not rise as much as the CR paradigm. No retention target or limit was set during this work; however, this has indicated that a monitoring system ensuring retention of a minimum number of windows per class may improve the paradigm's performance.

When comparing the approaches for rejection of the test set, it is observed that the CR paradigm significantly increases the accuracy in Figure 10. However, as shown in Table 4, the CR paradigm rejects a mean of 69.87% of the testing windows, whereas the QA and ENN paradigms reject 22.03% and 35.92% respectively. The Macro F1-scores suggest that the ENN and QA paradigms consistently perform better than the CR paradigm. The individual class scores also indicate generally that the CR paradigms class balance is worse, likely because the CR paradigm significantly rejects more of the classes it misclassifies. Given the function-driven nature of upper limb prostheses, a class balance is imperative to the controllability of the device. If over time a class performance degrades significantly, the device function is compromised. Furthermore, as suggested by Scheme et al., the window rejection rate should not be so high that it makes the system feel unresponsive (Scheme et al., 2013). Although offline testing was performed in this work and thus controllability could not be directly assessed, a ~70% window rejection is likely to cause the system's update rate to become inconsistent and fall below an acceptable level. This further indicates the QA and ENN paradigms may be more suitable than CR for rejection or that participant-specific rejection thresholds may need to be ascertained.

Comparison of post-processing rejection with the established majority vote technique showed that QA does not perform better than the standard method. As such, the QA paradigm may not be suitable for post-processing and should be prioritised for window-selection retraining. The ENN and CR paradigms outperform the majority vote, with CR improving approximately four times more than ENN. Whilst this suggests that there is potential for rejection paradigms to improve the magnitude of accuracy increase compared to majority vote, the controllability of a system employing majority vote is ensured by selecting a suitable number of neighbouring windows. As highlighted, rejection risks impacting the delay of the system updates if several windows are rejected sequentially, future online control tests would provide better indication of any benefit from rejection compared to majority vote.

In addition, when using the paradigms for rejection, a significant drawback of ENN is identified, which may limit its use in a clinical prosthesis. The ENN paradigm requires prior knowledge of the window's true class label, which is not possible during online operation. Adjustment of the paradigm to operate in an unsupervised manner would be necessary.

Finally, the runtimes of the three paradigms provide indication of suitability for use solely in embedded systems. The application of pattern recognition techniques to commercial prostheses is not commonly adopted due to computational complexity; therefore, paradigms should have a small computational cost. These are calculated as the mean of all retraining sessions, resulting in 0.048 ± 0.03 s for QA, 0.028± <0.01 s for CR, and 8.664 ± 2.07 s for ENN. It is apparent that the CR paradigm is the fastest, requiring only running the windows through the inference stage of the classifier. The QA paradigm is slightly slower, comparing the feature against a threshold. However, this does not account for the FFT calculation required. The FFT calculation on MATLAB of a single window took 350 us, increasing the paradigm runtime by ~25 s. However, an optimised digital signal processing block could be used to reduce this in an embedded system. The ENN paradigm is significantly slower, as the underlying KNN algorithm is slow, limiting its use solely on embedded control systems. Currently, this indicates that threshold-based paradigms, such as QA, may be more appropriate, despite a slight reduction in performance. Similar tests, performed on embedded hardware, would provide clearer indication of the suitability of each paradigm to a clinical application.

### 4.3 Future work

This work has compared paradigms across 10 recording sessions from 10 participants, using both the original Ninapro 6 dataset and an augmented version with added noise. As with rejection, future work could explore the use of participant-specific hyperparameters or thresholds for each of the paradigms, to further improve the individual classifier performances. However, the work presented here uses only offline data, as such a significant next step to demonstrating the effectiveness of the paradigms is to perform a study with online data.

In an online study, in addition to classification accuracy, metrics can be used to indicate the controllability of the system with and without retraining paradigms, such as monitoring the completion rate of tasks (Robertson et al., 2019). In turn, the use of these metrics can also help better tailor participant-specific thresholds. Furthermore, the recruitment of amputee participants is vital to show that performance improvements could be translated to the clinical domain. As highlighted, whilst the ENN paradigm achieved the greatest general performance, its application to online systems may be limited where true labels are required, such as in rejection. Therefore, future online testing could compare the paradigms with alternative retraining methods, such as the iterative online classifier presented by Huang et al. (2017).

In this study, each paradigm has been investigated individually to directly compare their performances and rates of retention. A potential avenue to improve overall performance could implement the fusion of two or all of the paradigms. Similarly to ensemble methods in machine learning, this fusion would use votes from each paradigm to determine whether a sample is retained for retraining. However, a fusion of the paradigms would increase computation during retraining. In addition, the implementation of retention targets or limits would need to be explored, to avoid class balance issues occurring from under- or over-retention.

Whilst this work has shown that the CNN is more suited to retaining, all cases using the LDA still significantly improved accuracy statistically against the baseline. Therefore, a traditional machine learning model that could be adapted to allow incremental training should be assessed similarly and may achieve fall-off behaviour and class balance similar to a CNN.

## 5 Conclusion

This study has presented a comparison of three paradigms designed to select the best windows from new datasets when adapting classifiers for sEMG pattern recognition. Adaption of the pattern recognition classifier is an important addition for sEMG control systems due to the non-stationary nature of the sEMG signal, which results in performance degradation between recording sessions.

The results of the study show that the use of Edited Nearest Neighbour provided the greatest significant improvement in accuracy for dataset selection on both the linear discriminant analysis and the convolutional neural network classifiers. However, drawbacks of the ENN were identified, requiring longer runtime and prior knowledge of the true class of extracted windows. These may limit its application in the clinical domain. Alternatively, the proposed Quality Assessment paradigm also achieved a similar improvement over the Confidence Retraining method, when only applied to retraining. This indicates that dataset selection may benefit from knowledge directly from the data, compared to the current classifier's exploration of it. The study investigated the paradigms handling of non-stationary sEMG data captured from 10 sessions across 5 consecutive days from the open NinaPro 6 dataset. Whilst achieving a lower starting accuracy than the LDA, the CNN was shown to maintain its accuracy better across the 10 sessions and in doing so retained a more balanced performance across the classes. The LDA, however, maintained its accuracy by improving its performance on the rest class at the detriment of the activation classes. Between individual sessions, the rest class will be more similar than the activations, as such these exemplars were often reinforced. As such, the use of iteratively trained classifiers such as CNN is recommended for retraining methods as they can more readily adjust to the underlying drift in the data.

## Data Availability

Publicly available datasets were analysed in this study. This data can be found at: the NinaPro repository http://ninapro.hevs.ch/, specifically DB6 presented in 10.1109/ICORR.2017.8009405.
